# Simplified dosimetry for kidneys and tumors in ^177^Lu-labeled peptide receptor radionuclide therapy

**DOI:** 10.1186/s40658-022-00473-z

**Published:** 2022-06-20

**Authors:** Oscar Ardenfors, Joachim N. Nilsson, Daniel Thor, Cecilia Hindorf

**Affiliations:** 1 Department of Radiation Physics and Nuclear Medicine, Karolinska University Hospital, 171 76 Stockholm, Sweden; 2 Department of Oncology-Pathology, Karolinska Institutet, Stockholm, Sweden; 3 Department of Molecular Medicine and Surgery, Karolinska Institutet, Stockholm, Sweden

**Keywords:** Kidney dosimetry, Tumor dosimetry, DOTATATE, Single-point dosimetry

## Abstract

**Purpose:**

To evaluate if satisfactory post-therapeutic image-based dosimetry can be achieved for Lu-177-DOTATATE treatments using a reduced number of image acquisitions to improve patient comfort and reduce economical costs.

**Methods:**

39 patients who underwent 147 treatment cycles of Lu-177-DOTATATE for neuroendocrine tumors were included in the study. A total of 291 and 284 absorbed doses were calculated to kidneys and tumors, respectively. Single-point dosimetry was performed using one SPECT/CT image acquired at 1 d or 7 d post-treatment using a fixed effective half-life (*T*_eff_) or using a patient-specific *T*_eff_ determined for the initial cycle. Also, dose-per-activity values, (*D*/*A*)_1_, were determined from the first cycle and used to calculate doses for subsequent cycles. All absorbed doses were evaluated against “true” doses calculated using both the 1 d and 7 d images. The relation between tumor grade and absorbed doses was also investigated. All dosimetry was performed on SPECT images.

**Results:**

Absorbed doses to kidneys were most accurate when single-point dosimetry was performed using 1 d images with median ratios in relation to “true” doses in total dose of 1.00 (IQR: 0.97–1.03) when using fixed *T*_eff_ and 1.01 (IQR: 0.98–1.04) when using *T*_eff_ from the initial cycle. Calculations based on the 7 d image were most accurate for tumors with corresponding ratios in total absorbed dose of 0.98 (IQR: 0.96–1.00) and 1.00 (IQR: 0.99–1.01) when using a fixed *T*_eff_ or *T*_eff_ from the first cycle, respectively. The (*D*/*A*)_1_ approach performed worse, as 2 of 77 total absorbed doses to the kidneys deviated with > 30%, and tumor-absorbed doses were increasingly overestimated with every cycle. Absorbed doses, *T*_eff_ and 1 d uptake were higher for G1 tumors than G2 tumors.

**Conclusion:**

Dosimetry can be performed with satisfactory accuracy when using single SPECT/CT images acquired at 1 d for kidneys or at 7 d for tumors.

## Introduction

The value of individualized dosimetry when treating neuroendocrine tumors (NET) using peptide receptor radionuclide therapy (PRRT) has been highlighted over the last years as ^177^Lu-DOTATATE has become commercially available as Lutathera^®^ [1–3]. One advantage of using ^177^Lu-DOTATATE in comparison to using PRRT with ^90^Y is the associated decrease in renal toxicity [3–9]. The reduction in risk of toxicity implies that the need for individualized kidney dosimetry could be much lower in PRRT delivered with ^177^Lu-DOTATATE and that it could even be considered as optional [2]. One of the disadvantages in omitting individualized kidney dosimetry is that the cumulated kidney dose would not be available when evaluating the patient status after the initial four standard treatment cycles possibly discouraging additional treatment cycles. Also, the lack of future dosimetry data would hamper the possibility to study the dose–response relationships associated with ^177^Lu-DOTATATE [1, 3, 10].

Considering the advantages and disadvantages associated with individualized dosimetry for ^177^Lu-DOTATATE, a simplified imaging schedule could offer a reasonable compromise without fully omitting patient dosimetry. Such compromise could be beneficial both from an economical and patient well-being point-of-view. Previous studies have shown that one or two image acquisitions can be sufficient to achieve a satisfactory accuracy in ^177^Lu-PRRT dosimetry [11–17]. It has been suggested that if a full dosimetry protocol is employed for the initial treatment cycle, single-point dosimetry can be carried out for subsequent cycles using effective half-lives determined from the first cycle as kidney effective half-lives are expected to stay relatively constant over the course of treatment [16, 18]. It has also been proposed that a single measurement performed at 4 d post-treatment is sufficient to perform renal and tumor dosimetry [11] while others claim that single-point dosimetry alone of the kidneys can be unreliable when using either SPECT/CT or planar imaging [14].

Image-based tumor dosimetry is not routinely performed for patients who undergo ^177^Lu-DOTATATE. One reason for this is that patient images are mostly acquired with SPECT/CT having a limited axial coverage of typically 40–50 cm which do not always include all tumor burden and both kidneys. Also, there is currently no consensus in how the tumor-absorbed doses should be clinically implemented [1, 3]. However, as more studies on tumor doses and dose–response relationships are being published there is reason to expect a future increase in clinical interest of tumor doses [3, 19, 20].

In this work, absorbed doses following ^177^Lu-DOTATATE therapy were calculated for each treatment cycle for kidneys and tumors. The accuracy in calculating absorbed doses using single-point dosimetry with SPECT/CT images acquired at 1 d or 7 d post-treatment was evaluated and compared with the accuracy in calculating absorbed doses for subsequent treatment cycles using administered activities and patient-specific dose-per-activity coefficients derived for the first treatment cycle. Patients were only imaged with SPECT/CT, removing uncertainties associated with dosimetry performed on planar images.

## Material and methods

### Patient data

A total of 39 patients diagnosed with NET and treated with ^177^Lu-DOTATATE PRRT were included in this retrospective study (written consent from all participants under Swedish ethical review authority approval nr. 2020-01,541). Each patient underwent between 2 and 8 treatment cycles adding up to a total of 147 treatment cycles resulting in 291 and 284 absorbed dose data points for kidneys and tumors, respectively. All patients were prescribed an activity of 7.4 GBq per treatment cycle. Patient characteristics are stated in Table 1. A minimum of five data points (absorbed doses to kidneys or tumors for a specific treatment cycle) were required for the corresponding treatment cycle to be included in the analysis. The evaluation of tumor dosimetry was carried out for all diagnoses combined and separately for small intestine NET with tumor grade G1 (9 patients) and G2 (16 patients) to investigate possible differences linked to tumor grade. Tumor grade was defined as G1 if Ki-67 indices in the most recent surgical specimen or biopsy material was < 3%, G2 for 3% ≤ Ki-67 ≤ 20% and G3 for Ki-67 indices > 20%. The differences between G1 and G2 with regard to absorbed dose, effective half-life and 1 d uptake were evaluated using a two-sample Student’s t-test with a significance level of 0.01.

**Table 1:** Patients characteristics [mean (range) where applicable]

Parameter	Value
No. of patients	39
Females	17
Males	22
Age	67 [35 88]
Diagnosis	
Small intestine NET	29
Pancreatic NET	5
Other NET	5
Grade	
G1	12
G2	23
G3	2
Unspecified	2
No. of treatment cycles	4 [2 8]
Administered activity (GBq)	7.6 [6.1 8.0]

### Image acquisition

For the first 15 patients (62 treatment cycles), SPECT/CT acquisitions were acquired at three timepoints: 1 d (28 h), 3 d and 7 d post-treatment. An in-house evaluation of these 15 patients performed in 2020 showed that only the early (1 d) and the late (7 d) SPECT/CT acquisitions were required to obtain a satisfactory dosimetry for kidneys and tumors. The ratio of absorbed doses calculated using two images and doses calculated using all three images were in good agreement with interquartile ranges (IQR) of 1.00–1.05 and 0.96–1.01 for kidneys and tumors, respectively. The feasibility of kidney dosimetry with one early and one late image has also been demonstrated by previous studies [12, 14]. Consequently, the 3 d acquisition was excluded from the clinical routine and only the 1 d and 7 d images were acquired for the subsequent patients. In order to keep a clean dataset, the 3 d images of the first 15 patients were excluded from the present study.

The upper abdomen, including kidneys, liver and spleen, was imaged. All SPECT images were acquired on a GE 670 Discovery system (International General Electric, General Electric Medical Systems, Haifa, Israel) equipped with a 3/8″ NaI(Tl) crystal and a medium energy general purpose collimator. The camera field-of-view (FOV) was 54 × 40 cm^2^ with a matrix size of 128 × 128 elements. Sixty projections per detector head were collected with an acquisition time of 20 s per projection. The primary energy window was centered at 208 keV ± 10.0% with upper and lower scatter windows centered at 168 keV ± 6.2% and 248 keV ± 4.2%, respectively. Images were reconstructed with the OSEM algorithm on a Xeleris workstation version 4.0 (International General Electric, General Electric Medical Systems, Haifa, Israel) using 6 iterations and 10 subsets. Resolution recovery and Butterworth post-filtering (cut off = 0.4, order = 5) were included in the reconstruction together with triple energy window scatter correction and CT-based attenuation correction. The CT was acquired using a low dose protocol with 120 kV tube voltage and 10 mAs tube current (with a pitch of 1.375 and rotation time of 0.5 s).

### Dosimetry

The reconstructed SPECT images were converted into units of activity using a sensitivity factor determined from phantom measurements using a methodology similar to that described by Marin et al. [21]. All dosimetry was carried out on patient images using an in-house software based on MATLAB. Activity concentrations at 1 d and 7 d were determined in accordance with the clinical routine by placing multiple VOIs (0.6 ml) within a homogenous uptake region of the medulla/cortex of the kidneys and different tumor volumes. The number of VOIs depended on size and activity homogeneity in the kidneys and tumors. The rationale behind using multiple small VOIs is to get a better estimate of the average dose to the whole organ/tumor in comparison to using a single VOI. Absorbed doses were calculated separately for the left and right kidney (one patient with only one kidney). The tumor volumes were included if the shortest diameter in any direction was at least 22 mm when a threshold based region-of-interest (42% threshold) was drawn and if the tumors could be tracked between treatment cycles [19]. A maximum of three tumor volumes per patient were included.

Cumulated activity concentrations, $\tilde{A}_{{\text{c}}}$, were calculated according to Eq. 1, in which the effective half-life, $T_{{{\text{eff}}}}$, was estimated assuming a mono-exponential excretion between 1 and 7 d, and the activity concentration, $A_{{{\text{c}},0}}$, at time $t = 0$, was calculated by decay correcting the activity concentration at 1 d using the corresponding $T_{{{\text{eff}}}}$. Absorbed doses, *D*, were then calculated using Eq. 2, where *ACDF* is an activity–concentration dose factor in units of mGy × cm^3^/MBq × s determined from validated in-house Monte Carlo simulations of ^177^Lu in water with MCNP6 [22]. The principle behind calculating absorbed doses using activity concentrations and *ACDF* while neglecting cross-firing is common for ^177^Lu and has been described elsewhere [23]. Absorbed doses determined using the 1 d and the 7 d images were calculated for all treatment cycles. These absorbed doses are denoted “true” absorbed doses henceforth:

1 $$\tilde{A}_{{\text{c}}} = \mathop \smallint \limits_{0}^{\infty } A_{{\text{c,0}}} \times \exp \left[ { - \frac{\ln \left( 2 \right) \times t}{{T_{{{\text{eff}}}} }}} \right]{\text{d}}t = A_{{\text{c,0}}} \times \frac{{T_{{{\text{eff}}}} }}{\ln \left( 2 \right)},$$

2 $$D = \tilde{A}_{{\text{c}}} \times {{ACDF}}{.}$$

### Absorbed dose per activity

The absorbed doses to the kidneys and tumor volumes per MBq of administered activity for the first treatment cycle, (*D*/*A*)_1_, were determined for each patient using the 1 d and 7 d images. Absorbed doses for the subsequent treatment cycles, *D*_*i*_, were then calculated according to Eq. 3 where *A*_*i*_ is the activity administered for cycle *i*. The absorbed doses per treatment cycle and the total absorbed dose from all treatment cycles were evaluated against the “true” absorbed doses. Also, ratios between total absorbed doses from all treatment cycles and corresponding “true” total doses were calculated and used to evaluate the agreement using a paired-sample Student’s t-test with a significance level of 0.01:

3 $$D_{i} = \left( {D/A} \right)_{1} \times A_{i} .$$

### Single-point dosimetry

Effective half-lives were determined for kidneys and tumor volumes using the 1 d and 7 d images from the first treatment cycle. These effective half-lives were used to calculate absorbed doses for subsequent treatment cycles using only activity concentrations determined from the 1 d or the 7 d images assuming a mono-exponential decrease from the time of injection. Furthermore, absorbed doses were calculated for kidneys and tumor volumes for all treatment cycles using a fixed $T_{{{\text{eff}}}}$ of 52 h and 109 h, respectively, corresponding to the median values of the patient cohort. All absorbed doses were compared to the “true” absorbed doses. The distributions of total absorbed dose ratios were compared using a paired-sample Student’s t-test with a significance level of 0.01.

## Results

### Absorbed dose, effective half-life and 1 *d* uptake per treatment cycle

**Fig. 1:**
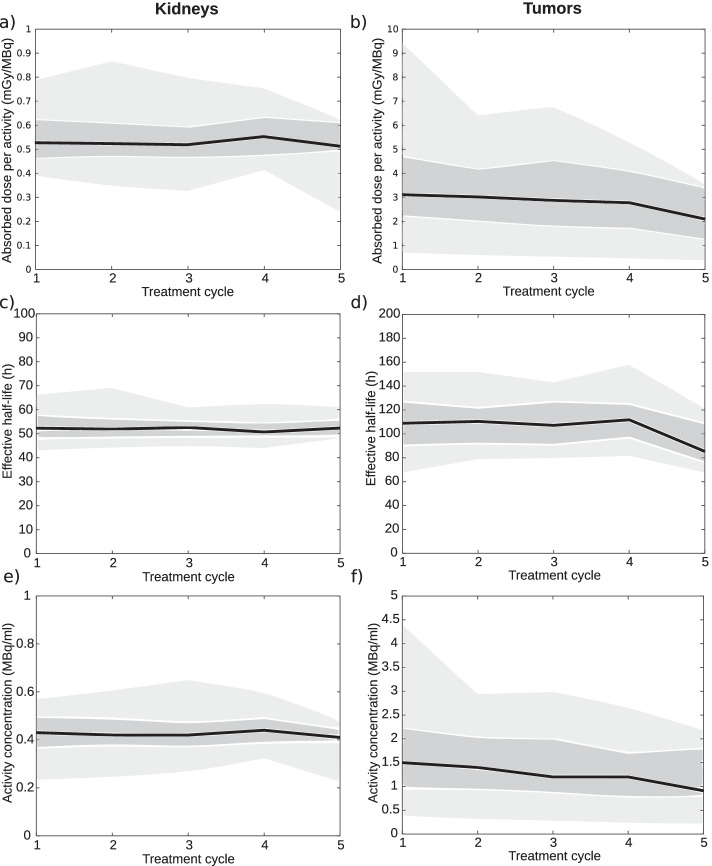
Median absorbed dose per administered activity per treatment cycle to the kidneys (**a)** and the tumors (**b)**. Median effective half-lives per treatment cycle to the kidneys (**c)** and the tumors (**d)**. Median uptake at 1 d per treatment cycle to the kidneys (**e)** and the tumors (**f)**. Shaded regions correspond to 25–75% percentile (dark grey) and 5–95% percentile (light grey)

The absorbed doses per administered activities, effective half-lives and activity concentrations at 1 d in the kidneys and the tumor volumes as function of treatment cycle for all patients included in the study are presented in Fig. 1. The absorbed doses and effective half-lives were calculated using both the 1 d and the 7 d SPECT/CT data. The results in Fig. 1 show that all kidney parameters were relatively constant for all treatment cycles. Tumor absorbed doses per administered activities slowly decreased over the first four treatment cycles after which a more prominent decrease of 0.7 mGy/MBq in median value was observed between the fourth and fifth treatment cycle. The tumor effective-half-lives were rather constant over the first four cycles after which a decrease of 26.7 h in median value was observed. The 1 d uptake in the tumors slowly decreased between treatment cycles and the difference between the first and fifth treatment cycle was 0.6 MBq/ml. The spread in absorbed dose to the kidneys was lower in comparison to the tumors as the corresponding normalized standard deviations were 28% and 75%, respectively. The analysis of small intestine NET grade showed that the tumor-absorbed doses per administered activity, effective half-lives and uptake at 1 d followed the same trend as for the combined tumors presented in Fig. 1. However, as presented in Table 2, absorbed doses, effective half-lives and 1 d uptakes were higher for G1 tumors for all treatment cycles.

**Table 2:** Median values with interquartile ranges for small intestine NET

Cycle	Absorbed dose (Gy)			Effective half-life (h)			1 *d* uptake (MBq/ml)		
	G1	G2	*p*	G1	G2	*p*	G1	G2	*p*
#1	30 (23–36)	18 (10–25)	1.85E-03*	118 (107–131)	102 (84–123)	2.48E-02	1.6 (1.3–2.1)	1.0 (0.8–1.4)	1.33E-02
#2	29 (21–38)	19 (10–23)	1.54E-03*	118 (107–123)	103 (91–120)	4.11E-02	1.6 (1.4–2.3)	1.2 (0.7–1.5)	7.83E-03*
#3	31 (19–45)	16 (8–24)	8.70E-04*	131 (114–141)	102 (88–120)	9.42E-04*	1.6 (1.2–2.1)	0.9 (0.5–1.5)	1.23E-02
#4	30 (19–34)	18 (10–26)	6.47E-03*	114 (107–125)	113 (99–133)	7.71E-01	1.7 (1.1–1.9)	0.9 (0.6–1.4)	6.52E-03*

*Significant difference between G1 and G2 (*p* < 0.01) in two-sample Student’s *t*-test

### Simplified dosimetry using absorbed dose per activity

**Fig. 2:**
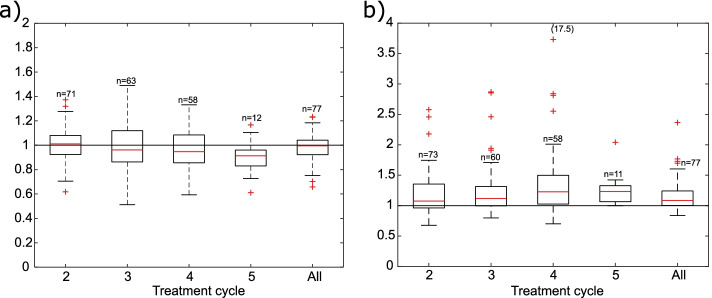
Normalized absorbed doses to kidneys (**a)** and tumors (**b)** calculated with administered activities and dose-per-activity values from the first treatment cycle. Absorbed doses are normalized to the “true” absorbed doses, *n* corresponds to number of data points included in the calculation for the corresponding treatment cycle. “All” refers to the total absorbed dose from all treatment cycles. A value of 17.5 for cycle 4 was cropped from Fig. 2b

Absorbed doses to the kidneys and the tumors relative to the “true” doses are presented in Fig. 2 for subsequent treatment cycles. The absorbed doses to the tumors relative to the “true” doses did not differ between tumor grades and results are therefore presented for all tumors combined. The median ratios and IQR of the total doses from all treatment cycles were 1.00 (IQR: 0.92–1.04) and 1.09 (IQR: 1.00–1.24) for the kidneys and tumors, respectively. The difference in distribution of total doses in relation to “true” total doses was significant (*p* < 0.01) for the tumors but not for the kidneys.

### Simplified dosimetry using single-point dosimetry

**Fig. 3:**
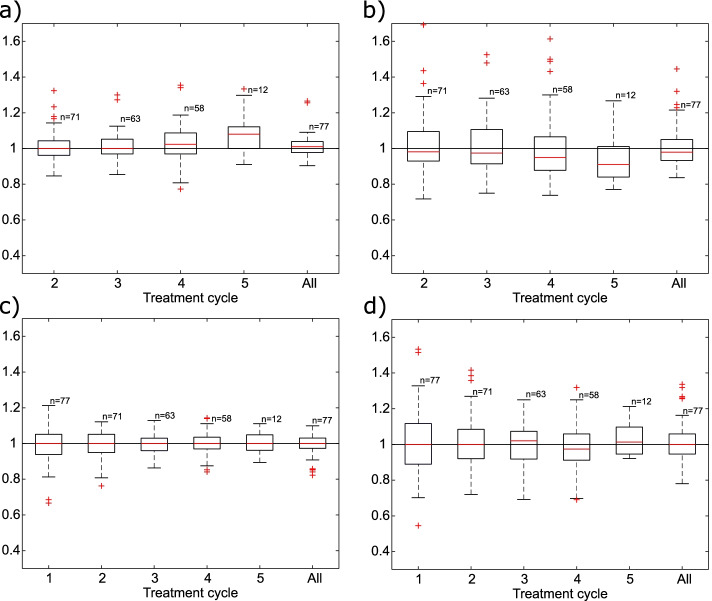
Absorbed doses to the kidneys normalized against “true” absorbed doses. Absorbed doses were calculated using the 1 d images and $T_{{{\text{eff}}}}$ from the first treatment cycle (**a)**, using the 7 d images and $T_{{{\text{eff}}}}$ from the first cycle (**b)**, using the 1 d images and a fixed $T_{{{\text{eff}}}}$ **c**, and using the 7 d images and a fixed $T_{{{\text{eff}}}}$ (**d)**. “All” refers to the total absorbed dose from all treatment cycles, *n* corresponds to number of data points included in the calculation for the corresponding treatment cycle

Relative absorbed doses to the kidneys calculated using the 1 d SPECT images or the 7 d SPECT images with effective half-lives determined from the first treatment cycle are presented in Fig. 3a, b. Corresponding single-point doses to the kidneys calculated with the fixed effective half-life of 52 h are presented in Fig. 3c, d. In general, the absorbed doses were in better agreement with the “true” doses when using the 1 d images as demonstrated by the smaller boxes corresponding to the IQR in Fig. 3a and c in comparison to Fig. 3b and d. It can be seen in Fig. 3c that the total absorbed dose to the kidneys were within 20% in relation to the “true” absorbed dose for all 77 kidneys when using the 1 d SPECT images and the fixed effective half-life to calculate the absorbed doses. The overall agreement in total dose to the kidneys was similar for all approaches with median ratios and IQR of 1.01 (IQR: 0.98–1.04), 0.98 (IQR: 0.93–1.05), 1.00 (IQR: 0.97–1.03) and 1.00 (IQR: 0.95–1.06) for the approaches presented in Fig. 3a–d, respectively. The Student’s *t*-test did not result in a significant difference in total absorbed doses in relation to the “true” total doses for any of the calculation approaches for kidneys.

**Fig. 4:**
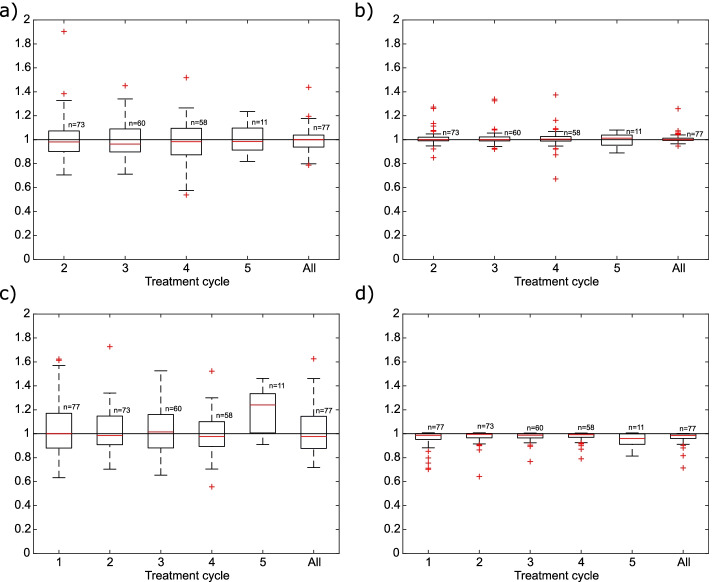
Absorbed doses to the tumors normalized against “true” absorbed doses. Absorbed doses were calculated using the 1 d images and $T_{{{\text{eff}}}}$ from the first treatment cycle (**a)**, using the 7 d images and $T_{{{\text{eff}}}}$ from the first cycle (**b)**, using the 1 d images and a fixed $T_{{{\text{eff}}}}$ (**c)**, and using the 7 d images and a fixed $T_{{{\text{eff}}}}$ (**d)**. “All” refers to the total absorbed dose from all treatment cycles, *n* corresponds to number of data points included in the calculation for the corresponding treatment cycle

Relative absorbed doses to the tumors calculated using single-point dosimetry are presented in Fig. 4. The absorbed doses were more accurate when using the 7 d images in comparison to the 1 d images with the best agreement found for the 7 d images together with the effective half-life determined from the initial cycle (76 out of 77 total doses were within 7%). The median ratios and IQR in total absorbed doses were 1.00 (IQR: 0.94–1.04), 1.00 (IQR: 0.99–1.01), 0.98 (IQR: 0.88–1.15) and 0.98 (IQR: 0.96–1.00) for the approaches in Fig. 4a–d, respectively. The separate results for the G1 and G2 tumors were similar to the trends for all tumors combined presented in Fig. 4 and are therefore not shown. No significant difference in distribution of total absorbed doses was found except for the 7 d images together with the fixed effective half-life for which the difference in relation to the “true” total doses was significant (*p* < 0.01).

## Discussion

The results of this study confirmed previous findings that kidney-absorbed doses are relatively constant between treatment cycles, whereas tumor doses slowly decrease with every treatment cycle for ^177^Lu-DOTATATE therapy [18, 20]. The spread in absorbed doses was found to be much larger for tumors: one normalized standard deviation of the absorbed doses was 28% for kidneys and 75% for tumors. It was also found that absorbed doses, effective half-lives and uptake at 1 d were higher for tumors in patients diagnosed with small intestine NET G1 in comparison to G2. This is in line with the results of Roth et al. [20] who reported higher absorbed doses to G1 tumors when observing a dataset of tumors with different origins combined (pancreas, small intestine, lung and unknown). Roth et al. reported a larger decrease in absorbed dose to G2 tumors for every treatment cycle in comparison to G1 tumors which was not observed for the small intestine NET in the present study. No difference in dosimetric accuracy due to tumor grading was found for any of the approaches investigated in the present study.

As the kidney-absorbed doses were rather constant and the tumor-absorbed doses decreased with every treatment cycle, the dose-per-activity approach performed better for kidneys than for tumors, for which the absorbed dose was increasingly overestimated for each treatment cycle. For any of the investigated approaches in this study to be clinically implemented, the data points with the largest errors must be few and the errors must not be too large as this could result in serious adverse effects for such “outlier” patients [14]. Relatively large errors may be acceptable for single treatment cycles, but not when considering the total dose from all cycles. Thus, the dose-per-activity approach appear unfeasible also for kidneys as it resulted in 2 outliers out of 77 data points for which the total dose corresponded to only 66% and 70% of the “true” total dose. While the biological effects of such inaccuracies are difficult to quantify, such calculations cannot be considered reliable and are therefore not suitable for clinical implementation.

The most accurate single-point dosimetry protocol for kidneys was the 1 d SPECT images together with the fixed effective half-life of 52 h for which the maximum difference in total dose was − 18% in relation to the “true” dose. Interestingly, both approaches using the early SPECT images outperformed the approaches using the late SPECT images for the kidneys. A possible benefit of performing single-point dosimetry at 1 d post-injection is that the patient can undergo both treatment and imaging before leaving the hospital. Promising results for single-point dosimetry using early SPECT images have been reported before. Willowson et al. [16] reported an average deviation of 2% and a maximum difference of 45% when calculating doses using 1 d SPECT images with effective half-lives from the first cycle (corresponding average and maximum difference was 7% and 35% for all kidney data points in the present study). Also, Sundlöv et al. [15] reported average differences of 1 ± 17% (2 standard deviations) and − 2 ± 25% when using 1 d SPECT images and effective half-life from the first treatment cycle or a fixed effective half-life of 51.6 h. The corresponding values for all kidney data points in the present study were 7 ± 13% and 5 ± 10%, respectively.

For tumors, single-point dosimetry was most accurately performed using the late SPECT images. This is in agreement with Hänscheid et al. [11] who reported an increased accuracy in single-point dosimetry for tumors with increasing time post-treatment (Pearson correlation coefficient between approximated time integral and actual time integral of 0.99 at 144 h). It should be noted that the tumor-absorbed doses in the present study were significantly lower in comparison to the “true” doses when calculated using the late SPECT image together with the fixed effective half-life (Fig. 4). This indicates that, unlike for kidneys, single-point dosimetry for tumors is more accurate when using a tumor-specific effective half-life determined from the initial treatment cycle.

Single-point dosimetry for kidneys proved more accurate when using a fixed effective half-life of 52 h instead of a patient-specific value from the first treatment cycle or a dose-per-activity value determined from the first cycle. This could imply that the uncertainty in determining the effective half-life or dose-per-activity values for one treatment cycle is higher than the difference in kinetics between one individual patient and the population for which the fixed effective half-life was calculated. One drawback associated with the present study is that the “true” absorbed doses were calculated from only two data points (1 d and 7 d). Two data points are not sufficient to fully reproduce the associated kinetics of tumor volumes or kidneys, that are likely to behave more closely to a bi-exponential function. However, a third data point at, e.g., 4 d does not provide more information regarding the initial uptake but rather serves to improve the curve fit and reduce the impact from possible errors associated with the 1 d or 7 d image acquisitions (e.g., poor statistics, motion artifacts, etc.).

It should be noted that only SPECT/CT images were included in the present study removing all uncertainties related to the corrections needed when calculating absorbed doses from planar images. Also, scatter correction and CT-based attenuation correction were used on all images reducing potential errors associated with patient geometries. Regarding the small VOI approach used in the present study, it has been shown that absorbed doses can be systematically higher when calculated using maximum activity concentrations in the kidneys in comparison to using activities within a volume delineated on CT images [12]. This difference should however be smaller when using several VOIs throughout the kidneys as in the present study. The fixed effective half-lives of the kidneys and tumors used in the present study were chosen as the median values of all patients which may have limited generalization to other patient cohorts. However, the value of 52 h used for kidneys in the present study is in excellent agreement with published data [8, 14, 16, 18]. For tumors, less data are available for comparison and the variation in effective half-lives is expected to be larger. Roth et al. [20] reported average effective half-lives of 81 h and 103 h for G1 and G2 tumors, respectively. To assess the robustness of our single-point calculations, fixed effective half-lives were varied between 81 and 137 h. This resulted in median values of the total normalized absorbed doses (7 d images) of 1.06 (IQR: 1.04–1.07), 0.98 (IQR: 0.96–1.00) and 0.99 (IQR: 0.97–1.00) for fixed effective half-lives of 81 h, 109 h and 137 h, respectively. This indicates that the calculations are not sensitive to moderate variations in tumor effective half-lives.

In agreement with other studies [12, 14], our previous in-house evaluation showed that when reducing the number of SPECT images from three (1 d, 3 d and 7 d) to two acquisitions, the best accuracy was obtained when using the 1 d and 7 d images. Previous work on single-point dosimetry by Hänscheid et al. [11] suggests that absorbed doses can be determined with satisfactory accuracy for tumors and kidneys when using an image acquired 4 d post therapy. An optimal timepoint for kidneys at 4 d post therapy was also suggested by Sundlöv et al. [15]. When considering these findings together with the results of the present study it could be argued that if single-point dosimetry is to be performed, the images should be acquired at 1 d or 4 d if kidney doses are of interest, 4 d or 7 d if tumor doses are of interest, and at 4 d if both kidney and tumor doses are of interest. This suggests that the imaging schedule can be adjusted to the logistical reality of the clinic within this timeframe without compromising much accuracy in dosimetry.

In conclusion, our results show that single-point dosimetry is feasible for both kidneys and tumors. However, the 1 d SPECT image proved to be optimal for kidneys and the 7 d SPECT proved to be optimal for tumors (independent of tumor grading).

## Data Availability

Data can be made available after contacting the authors.
